# Two hearts, one fear? Dyadic fear-of-progression and quality of life among Thai gynecologic-cancer survivors and caregivers

**DOI:** 10.3389/fpsyg.2025.1640178

**Published:** 2025-11-10

**Authors:** Nutthaporn Chandeying, Therdpong Thongseiratch

**Affiliations:** 1 Faculty of Medicine Vajira Hospital, Bangkok, Thailand; 2 Faculty of Medicine Ramathibodi Hospital, Mahidol University, Bangkok, Thailand

**Keywords:** fear of progression, fear of recurrence, gynecologic cancer, survivors, caregivers, quality of life, Thailand

## Abstract

**Objective:**

To examine actor-partner interdependence between fear-of-progression (FoP) and global quality of life (QOL) in Thai gynecologic-cancer survivor–caregiver dyads.

**Methods:**

A cross-sectional study recruited 300 survivor–caregiver pairs from tertiary oncology centers in Bangkok, Thailand. Survivors were ≥6 months post-treatment for cervical, ovarian, or uterine cancer. Dyads completed the Thai Fear of Progression Questionnaire Short Form and the WHOQOL-BREF. Actor–Partner Interdependence Models (APIM) were estimated with structural equation modeling, treating dyad members as distinguishable (patient vs. caregiver). Models controlled for age, time since diagnosis, and comorbidity count.

**Results:**

Mean FoP scores were 27.4 ± 9.3 for survivors and 26.8 ± 8.8 for caregivers; mean QOL totals were 88.9 ± 12.1 and 90.2 ± 12.4, respectively. FoP levels were moderately correlated within dyads (r = 0.37, *p* < 0.001). In APIM, higher FoP predicted poorer QOL for the same person (actor effects: *β* = −0.38, *p* < 0.001 for survivors; β = −0.25, *p* = 0.001 for caregivers). Partner effects were small and non-significant (caregiver FoP → survivor QOL: *β* = −0.03, *p* = 0.46; survivor FoP → caregiver QOL: β = −0.05, *p* = 0.28). Goodness-of-fit indices supported the actor-only pattern (χ^2^ = 3.4, df = 4, *p* = 0.49; RMSEA = 0.00; CFI = 1.00).

**Conclusion:**

Among Thai gynecologic-cancer dyads, fear-of-progression erodes the individual’s own quality of life but does not appear to does not appear to exert a cross-partner influence. Psycho-oncology programs should therefore screen and treat FoP in both survivors and caregivers, yet expect QOL gains to arise chiefly from direct, rather than cross-partner, relief of fear. Because the design was cross-sectional, temporal ordering cannot be inferred; FoP–QOL associations may be bidirectional (e.g., poorer QOL amplifying FoP and vice versa). Longitudinal, multi-wave APIM is needed to establish directionality. Future work should test domain-level QOL outcomes and longitudinal APIM to determine whether subtle cross-partner effects emerge in specific life domains.

## Introduction

Gynecologic malignancies collectively account for more than 1.3 million new cases and over 600,000 deaths each year worldwide, making them a major contributor to the global cancer burden despite advances in screening, surgery, radiotherapy, and systemic therapies (Sung et al., 2021). The shift from acute treatment to long-term survivorship has highlighted psychosocial sequelae that accompany improved survival, one of the most pervasive being Fear of Progression (FoP), the persistent worry that disease will advance or treatments will intensify, even if remission has not yet been achieved (Coutts-Bain et al., 2022). Conceptually distinct from Fear of Cancer Recurrence (FCR), FoP encompasses concerns about functional decline, disability, and future medical procedures (Coutts-Bain et al., 2022; Herschbach et al., 2005). Systematic reviews indicate that 40 –70% of gynecologic-cancer survivors report clinically relevant FoP, a prevalence that often exceeds depressive or post-traumatic stress symptoms in this population (Mehnert et al., 2006; Koch et al., 2013). Elevated FoP has been linked to poorer adherence to surveillance schedules, greater health-care utilization, and diminished global quality of life (QOL) (Mutsaers et al., 2016; Dinkel et al., 2021; Thewes et al., 2016). Nevertheless, oncologic follow-up clinics seldom include FoP screening, and most psychosocial interventions remain in exploratory phases (Zhang et al., 2022).

Family caregiving is a universal phenomenon in oncology and introduces a second locus of psychological distress. Spouses, adult children, and siblings frequently assume roles that encompass emotional support, treatment coordination, and financial management (Northouse et al., 2010). Systematic reviews show that caregivers experience levels of anxiety, depressive symptoms, and FoP comparable to, or even surpassing, those of the survivors for whom they care (Kim and Schulz, 2008; Fletcher et al., 2012). High caregiver distress has been correlated with reduced patient adherence, greater decisional regret during therapy selection, and increased unplanned hospital admissions (Applebaum and Breitbart, 2013; Baik and Adams, 2011; Porter et al., 2009). Beyond simple correlation, the stress-process model posits bidirectional influences within dyads: one member’s distress can exacerbate the other’s symptom burden, while satisfactory coping in either partner may buffer global well-being for both (Kenny et al., 2006). Despite this interdependence, most quantitative studies still analyse survivors and caregivers as independent units, thereby overlooking cross-partner dynamics that might inform family-based interventions (Regan et al., 2014).

The actor–partner interdependence model (APIM) offers a robust analytic framework for disentangling such dyadic processes. APIM simultaneously estimates how a predictor in one partner influences their own outcome (actor effect) and their counterpart’s outcome (partner effect), while accounting for within-pair non-independence (Pietromonaco et al., 2013). Studies applying APIM to breast, colorectal, and prostate cancer have revealed consistent actor effects for anxiety and depression and occasional partner effects for coping style, communication, and decision satisfaction (Peikert et al., 2020). However, very few APIM investigations have centred on FoP, and those that do exist have focused primarily on parents of childhood-cancer survivors or on couples coping with breast cancer (Christakis and Allison, 2006). To date, no large-scale APIM study has examined adult gynecologic-cancer survivors paired with their primary family caregivers, leaving a crucial gap in the psycho-oncology literature.

Cross-cultural research further suggests that caregiving norms, health-system characteristics, and gender roles can modulate dyadic stress pathways (Chatters et al., 2015). For instance, collectivist societies may experience stronger emotional contagion within families, whereas individualist contexts might foster more independent coping strategies. Analyses from multinational samples emphasize that partner effects are not uniform; they may vary by cancer stage, relationship quality, and socioeconomic status (Manne and Badr, 2008; Hasson-Ohayon et al., 2018; Badr and Krebs, 2013). Moreover, gynecologic cancers uniquely affect sexuality, fertility, and body image, factors that can strain intimate partnerships and magnify caregiving burden irrespective of cultural setting. A dyadic examination of FoP in this disease group could therefore uncover actionable patterns that transcend national borders, informing universal and locally adaptable interventions.

The present study investigates actor and partner associations between FoP and global QOL in survivor–caregiver dyads affected by cervical, ovarian, or uterine cancer. We hypothesised that higher FoP would predict poorer QOL for the same individual (actor effects) and exert additional cross-partner influences on the partner’s QOL (partner effects). By clarifying these pathways in a robust sample of gynecologic-cancer dyads, our work aims to advance theoretical understanding of family adjustment to cancer and to guide the development of evidence-based, dyad-focused psychosocial care.

## Materials and methods

### Study design and participants

We conducted a cross-sectional dyadic survey in Bangkok tertiary gynecologic-oncology centers between January and June 2023. Consecutive adult survivors (≥ 18 years) of cervical, ovarian, or uterine cancer who were ≥ 6 months post-completion of primary treatment were invited during routine follow-up visits. Each survivor nominated one primary family caregiver who self-identified as providing the greatest unpaid support. Exclusion criteria were metastatic relapse under active treatment, severe cognitive impairment, or inability to provide informed consent. Ethical approval was obtained from the institutional review board (COA 178/62), and all participants gave written informed consent in accordance with the Declaration of Helsinki.

### Measures

#### Fear of progression (FoP)

Survivors and caregivers completed the 12-item Fear of Progression Questionnaire Short Form (FoP-Q-SF), which has demonstrated robust reliability and factorial validity across cancer populations (Cronbach’s *α* ≈ 0.86–0.91) (Herschbach et al., 2005). Items are rated 1 (never) to 5 (very often); summed scores range 12–60, with higher scores indicating greater FoP. Caregivers completed the validated partner/relative version of the short form (FoP-Q-SF/PR; 12 items), which mirrors the patient version but references caregiving concerns rather than personal illness (e.g., “I worry about the disease progressing” phrased with respect to the survivor). Item anchors, scoring range (12–60), and interpretation are identical to the patient FoP-Q-SF.

#### Quality of life (QOL)

Global QOL was assessed with the WHOQOL-BREF, a 26-item instrument derived from the WHOQOL-100 that shows cross-cultural measurement invariance and sound psychometrics (α ≈ 0.90) (The WHOQOL Group, 1998). Each item is scored 1–5; the raw total (26–130) was used as the outcome variable. Because our *a priori* objective was to characterize overall well-being at the dyadic level, we analyzed the WHOQOL-BREF total score.

#### Covariates

Survivors reported age, cancer type, stage at diagnosis, time since diagnosis, and comorbidity count (Charlson index). Caregivers reported age, relationship to survivor, and weekly caregiving hours.

### Procedure

Eligible dyads completed paper questionnaires separately in a quiet clinic room, supervised by trained research nurses. Completed forms were double-entered into EpiData 3.1 with 10% random verification; any inconsistencies were resolved against the original source documents. Item-level missingness was < 2% and was handled with full-information maximum likelihood (FIML) during modeling.

### Statistical analysis

Analyses were performed in R 4.3.2 using the *lavaan* package. Descriptive statistics summarized sample characteristics. Between-role differences in FoP and QOL were examined with paired *t*-tests; intraclass correlations quantified within-dyad similarity.

APIM were tested with structural-equation modelling, treating survivors and caregivers as distinguishable roles. In practical terms, the analysis asked four linked questions at once: (1) Does a survivor’s fear of progression (FoP) predict their own quality of life (actor effect)? (2) Does a survivor’s FoP exert a cross-partner influence on the caregiver’s quality of life (partner effect)? (3) Does a caregiver’s FoP affect their own quality of life (actor effect)? and (4) Does a caregiver’s FoP exert a cross-partner influence on the survivor’s quality of life (partner effect)? All paths were adjusted for survivor age, time since diagnosis, and comorbidity count, as well as caregiver age, to minimise confounding (Kline, 2016).

Model performance was judged by widely accepted goodness-of-fit criteria: a non-significant chi-square test, comparative-fit index (CFI) of 0.95 or higher, root-mean-square error of approximation (RMSEA) of 0.06 or lower, and standardized root-mean-square residual (SRMR) below 0.08. Finally, we used bias-corrected bootstrap samples to generate robust standard errors and 95% confidence intervals, providing more reliable estimates even with slight departures from normality (Ledermann et al., 2011).

Models were estimated using full-information maximum likelihood (FIML) under a Missing At Random (MAR) assumption. MAR is plausible given the low rate of missingness and patterns primarily related to observed covariates (e.g., age, months since diagnosis, caregiving hours), which we included as auxiliary variables in the SEM to enhance parameter recovery when missingness depends on observed data.

## Results

### Sample characteristics

A total of 300 survivor–caregiver dyads were enrolled; item-level missingness was < 2% and handled with FIML, yielding an effective analytic sample of 300 pairs for descriptive analyses and 292 pairs for the APIM (those with complete covariate data). Survivors were a median 52 years old (IQR 45–60) and a median 26 months post-diagnosis, while caregivers were a median 44 years old (IQR 36–54) and most frequently spouses (36%). Detailed socio-demographic and clinical characteristics are presented in Table 1.

**Table 1:** Socio-demographic and clinical characteristics of gynecologic-cancer survivors and their family caregivers (*N* = 300 dyads).

Variable	Survivors (*n* = 300)	Caregivers (*n* = 300)
Age, y	52.4 ± 13.0 (mean ± SD)	43.9 ± 13.1
Sex	Female 300 (100%)	Male 143 (47.7%) Female 146 (48.7%) Missing 11 (3.7%)
Marital status	Single 68 (22.7%) Married 197 (65.7%) Divorced/Widowed 32 (10.7%) Missing 3 (1.0%)	Single 93 (31.0%) Married 182 (60.7%) Divorced/Widowed 21 (7.0%) Missing 4 (1.3%)
Relationship to survivor	Not applicable	Spouse 108 (36.0%) Parent 33 (11.0%) Other relative 116 (38.7%) Other 39 (13.0%) Missing 4 (1.3%)
Religion	Buddhist 285 (95.0%) Christian 2 (0.7%) Islam 13 (4.3%)	Buddhist 280 (93.3%) Christian 2 (0.7%) Islam 13 (4.3%) Other/unspecified 5 (1.7%)
Education level	No formal schooling 11 (3.7%) Primary 105 (35.0%) Lower secondary 48 (16.0%) Upper secondary/vocational 49 (16.3%) Associate/diploma 22 (7.3%) Bachelor or higher 61 (20.3%) Missing 4 (1.3%)	No formal schooling 2 (0.7%) Primary 44 (14.7%) Lower secondary 46 (15.3%) Upper secondary/vocational 62 (20.7%) Associate/diploma 22 (7.3%) Bachelor or higher 120 (40.0%) Missing 4 (1.3%)
Main occupation	Housewife 93 (31.0%) Employee/business/government 135 (45.0%) Retired 14 (4.7%) Unemployed 39 (13.0%) Other 17 (5.7%) Missing 2 (0.7%)	Housewife 25 (8.3%) Employee/business/government 198 (66.0%) Retired 17 (5.7%) Unemployed 23 (7.7%) Other 31 (10.3%) Missing 6 (2.0%)
Annual income, median [IQR], USD*	2,750 [490–5,615]	4,120 [880–6,885]
Has personal savings	120 (40.0%)	138 (46.0%)
Cancer type	Cervical 102 (34.0%) Ovarian 98 (32.7%) Endometrial/uterine 81 (27.0%) Other gynecologic 19 (6.3%)	Not applicable
Time since diagnosis, mo	26 [14–48] (median [IQR])	Not applicable
Comorbid conditions†	Hypertension 104 (34.7%) Hyperlipidemia 69 (23.0%) Diabetes 46 (15.3%) Heart disease 7 (2.3%) Kidney disease 9 (3.0%)	Hypertension 53 (17.7%) Hyperlipidemia 36 (12.0%) Diabetes 28 (9.3%) Heart disease 6 (2.0%) Kidney disease 3 (1.0%)

Values are *n* (%) unless otherwise specified. Percentages are calculated from non-missing data within each role. Missing values are listed explicitly as “Missing *n* (%).” Continuous variables are mean ± SD or median [IQR] as indicated. * Converted from Thai baht at 36 THB ≈ 1 USD for descriptive purposes.

† Self-reported physician diagnoses; categories are not mutually exclusive. Percentages are calculated from non-missing data within each role; totals may not equal 100% due to rounding.

### Descriptive statistics and within-dyad correlations

As shown in Table 2, mean FoP-Q-SF scores were 27.4 ± 9.3 for survivors and 26.8 ± 8.8 for caregivers (paired *t* = 1.02, *p* = 0.31). Mean WHOQOL-BREF totals were 88.9 ± 12.1 and 90.2 ± 12.4, respectively (paired *t* = −1.42, *p* = 0.16). Intraclass correlations indicated moderate within-dyad similarity for FoP (ICC = 0.37, 95% CI 0.27–0.46) and QOL (ICC = 0.32, 95% CI 0.21–0.41). Zero-order Pearson correlations among study variables are provided in Table 3.

**Table 2:** Fear-of-progression and global quality-of-life scores in survivor–caregiver dyads (*N* = 300 dyads).

Measure (possible range)	Survivors (Mean ± SD)	Caregivers (Mean ± SD)	Mean difference* (caregiver − survivor)	*t*(df = 299)	*p*
FoP-Q-SF total (12–60)	27.4 ± 9.3	26.8 ± 8.8	−0.6 (95% CI − 1.8 to +0.6)	1.02	0.31
WHOQOL-BREF total (26–130)	88.9 ± 12.1	90.2 ± 12.4	+1.3 (95% CI − 0.5 to +3.1)	−1.42	0.16

**Table 3:** Pearson correlations among fear-of-progression (FoP), global quality of life (QOL) and selected covariates (*N* = 300 dyads*).

#	Variable	1	2	3	4	5	6	7	8	9
1	Survivor FoP (FoP-P)	—								
2	Caregiver FoP (FoP-C)	0.37*	—							
3	Survivor QOL (QOL-P)	−0.46*	−0.12	—						
4	Caregiver QOL (QOL-C)	−0.08	−0.29*	0.32*	—					
5	Survivor age, y	0.05	0.03	−0.18	−0.06	—				
6	Caregiver age, y	0.04	0.07	−0.09	−0.15	0.41***	—			
7	Months since diagnosis	*-0.13 **	−0.06	0.11	0.05	−0.03	—	—		
8	Weekly caregiving hours	0.08	0.21	−0.10	−0.24	0.02	0.12	0.06	—	
9	Survivor comorbidity count†	0.15*	0.05	−0.22	−0.09	0.18**	0.04	0.03	0.07	—

*Pairwise *n* = 300 (|r| ≥ 0.11 ≈ *p* < 0.05). †Number of physician-diagnosed chronic conditions (0–10). Significance: *p < 0.05; **p < 0.01; ***p < 0.001. Correlations confirm the patterns reported in the APIM: FoP within dyads is moderately concordant (r = 0.37) and each partner’s FoP is inversely related to their own QOL, whereas cross-partner associations are small and non-significant.

### Actor–partner interdependence model

**Figure 1:**
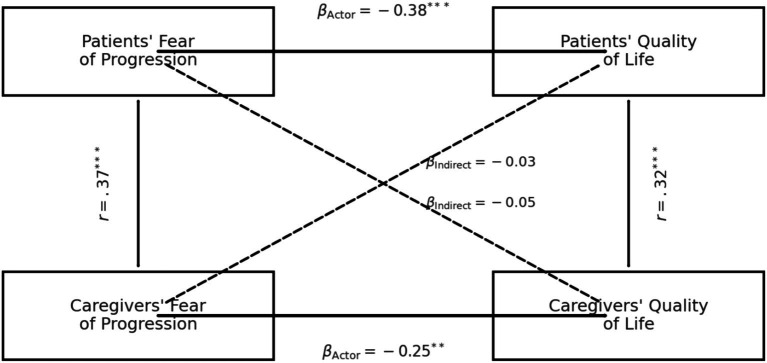
Actor-partner interdependence model (APIM).

Re-estimating models with complete cases (*n* = 292) yielded actor and partner coefficients that were within the FIML 95% CIs and did not change substantive conclusions. These findings indicate that our results are robust to reasonable assumptions about the missing-data mechanism (MAR) and to the choice of estimation (FIML vs. complete-case). The hypothesised APIM demonstrated excellent fit to the data (χ^2^ = 3.42, df = 4, *p* = 0.49; CFI = 1.00; RMSEA = 0.00; SRMR = 0.02). Standardised path coefficients are displayed in Figure 1 and numerically summarised in Table 4. Actor effects were significant for both dyad members: higher FoP predicted poorer QOL for survivors (*β* = −0.38, 95% CI –0.50 to −0.25, *p* < 0.001) and for caregivers (*β* = −0.25, 95% CI –0.39 to −0.11, *p* = 0.001). Partner effects were small and non-significant: caregiver FoP did not predict survivor QOL (β = −0.03, 95% CI –0.12 to 0.06, *p* = 0.46) and survivor FoP did not predict caregiver QOL (β = −0.05, 95% CI –0.15 to 0.05, *p* = 0.28). The partner-to-actor ratio (*k*) was 0.08 for the survivor equation and 0.20 for the caregiver equation, confirming an actor-only pattern.

**Table 4:** Standardised actor and partner effects from the actor–partner interdependence model (APIM) predicting global quality of life (WHOQOL total) from fear of progression (FoP) in gynecologic-cancer dyads (*n* = 292*).

Path	Standardised β	95% CI†	*p*
Actor effect – survivor (survivor FoP → survivor QOL)	−0.38	−0.50 to −0.25	< 0.001
Partner effect – survivor-directed (caregiver FoP → survivor QOL)	−0.03	−0.12 to +0.06	0.46
Actor effect – caregiver (caregiver FoP → caregiver QOL)	−0.25	−0.39 to −0.11	0.001
Partner effect – caregiver-directed (survivor FoP → caregiver QOL)	−0.05	−0.15 to +0.05	0.28

Model fit indices: χ^2^ = 3.42 (df = 4, *p* = 0.49); CFI = 1.00; RMSEA = 0.00; SRMR = 0.02. k-ratio (partner/actor): survivor equation = 0.08; Caregiver equation = 0.20—pattern consistent with an actor-only model. *Eight dyads omitted because of missing covariate data; full-information maximum-likelihood estimation used for the remaining 292 pairs. †Bias-corrected 5,000-bootstrap confidence intervals.

### Sensitivity analyses

Excluding 24 survivors with FIGO stage IV disease (*n* = 268 dyads) or grand-mean centring FoP scores produced virtually identical actor coefficients (β range −0.37 to −0.40) and left partner paths non-significant (all *p* > 0.25), reinforcing model robustness.

## Discussion

The present dyadic analysis investigated how fear of progression (FoP) relates to global quality of life (QOL) among survivors of gynecologic cancer and their family caregivers. Consistent with our first hypothesis, greater FoP was associated with poorer QOL for the individual reporting that fear; actor effects were moderate in survivors (β = −0.38) and small-to-moderate in caregivers (β = −0.25). Contrary to our second hypothesis, partner effects were negligible and non-significant. These findings delineate an actor-only pattern, echoing previous APIM studies in colorectal-cancer (Kim et al., 2025), childhood-cancer (Peikert et al., 2020), and lung-cancer dyads (Weng et al., 2023), yet contrast with investigations that observed modest partner pathways for dyadic coping, depression, or intimacy in mixed-cancer samples (Regan et al., 2014). Hence, FoP appears to erode the well-being of its bearer without exerting a cross-partner influence on the other member, even though FoP and QOL cluster within dyads. Our focal construct is FoP, concern about disease worsening, functional decline, and future treatments. FoP is related to but distinct from fear of cancer recurrence (FCR), which centers on return of disease after remission. We therefore interpret all findings in the context of FoP; references to the FCR literature are used only as construct-adjacent context (e.g., shared cognitive–affective mechanisms), not as evidence about FCR in our sample.

Several mechanisms may explain the predominance of actor effects. First, FoP is highly individualized—rooted in personal illness beliefs, prior traumatic memories, and perceived symptom fluctuations (Mutsaers et al., 2016; Dinkel et al., 2021). Qualitative data suggest that survivors and caregivers often internalize fears rather than openly discuss them, a coping strategy that limits direct emotional transmission (Thewes et al., 2016; Otto et al., 2016). Second, gynecologic cancers entail gendered and body-image concerns that survivors may selectively share with peers or clinicians rather than with caregivers, curbing partner influence. Third, caregivers in the current sample spent a median of 28 h per week in direct care, a burden that may leave little emotional “bandwidth” to absorb additional distress from survivors—an observation aligned with caregiver-strain models positing resource depletion and emotional numbing (Northouse et al., 2010; Wagner et al., 2006). Collectively, these factors likely reinforced self-focused rather than reciprocal pathways.

Several dyadic theories help explain limited partner effects. Dyadic coping and relationship processes suggest that crossover depends on intimacy, trust, and communication openness; when communication is constrained or problem-focused rather than emotion-focused, cross-partner transmission weakens [e.g., intimacy/communication frameworks in cancer couples (Manne and Badr, 2008; Badr and Krebs, 2013)]. Attachment and close-relationship perspectives posit that individual appraisals and regulation strategies (e.g., deactivating vs. hyperactivating) can localize distress within a person, curbing cross-partner pathways (Pietromonaco et al., 2013). Caregiver-strain models indicate that higher burden may blunt emotional responsiveness, limiting capacity to absorb a partner’s fear (Wagner et al., 2006). Family/systemic views and the “linked lives” principle emphasize shared context without requiring direct causal crossover—consistent with our moderate within-dyad clustering alongside negligible partner paths (Kenny et al., 2006; Elder et al., 2003). Together, these lenses imply that partner effects are contingent rather than universal. Partner pathways may be stronger when relationship quality and communication openness are high, but weaker under greater caregiver burden (time/role demands) that constrains responsiveness. Cultural norms around emotional disclosure—relevant in Thai family contexts and gynecologic-cancer care—may also favor self-containment over reciprocal sharing, attenuating cross-partner effects. Future studies should test these moderators with interaction or multi-group APIM.

Although our models showed excellent fit and adequate statistical power, several measurement-related issues may have attenuated partner pathways. First, global QOL totals, while psychometrically strong, aggregate across heterogeneous domains (physical, psychological, social, environment). Such aggregation can dilute relatively small, domain-specific crossover signals (e.g., a survivor’s FoP selectively undermining a caregiver’s psychological or social QOL). Second, role-linked reporting tendencies may have introduced asymmetric social desirability: survivors could under-report distress to avoid burdening caregivers, whereas caregivers may normalize strain as part of their role. Third, modest restriction of range in FoP (typical in outpatient survivorship cohorts) can reduce covariance available for cross-partner prediction. Together, these considerations suggest that the observed actor-only pattern may reflect both true self-focused effects of FoP and the limited sensitivity of global outcomes to detect subtle cross-partner effects.

Clinically, these data advocate routine FoP screening for both survivors and caregivers. The International Psycho-Oncology Society recommends distress screening as the “sixth vital sign” (Bultz and Carlson, 2006), yet caregiver implementation remains patchy. Our actor-only pattern implies that reducing FoP in one partner is unlikely to produce collateral gains in the other; therefore, interventions should include separate, role-specific modules. Cognitive-behavioral therapy targeting catastrophic thoughts, mindfulness-based stress reduction, and acceptance-and-commitment techniques have each demonstrated medium effect sizes for survivorship fears (Zhang et al., 2022). Few trials, however, enroll caregivers; early dyadic pilot work combining parallel survivor and caregiver group sessions reduced anxiety but did not significantly shift QOL, mirroring our data on limited cross-over effects (Otto et al., 2016). Future interventions might incorporate flexible, blended delivery (e.g., digital psycho-education plus brief in-person counselling) to address time constraints faced by caregivers (Wagner et al., 2006).

The actor-only pattern implies that reducing FoP chiefly benefits the treated individual, with little cross-partner influence. From a resource-allocation perspective—especially in settings with constrained psycho-oncology capacity—this favors individual-level first-line care (e.g., brief CBT/ACT or mindfulness modules, digital self-help with minimal guidance) over routine dyadic formats. A pragmatic stepped-care approach is suggested: (Step 1) low-intensity, individual interventions for survivors and caregivers separately; (Step 2) escalate to therapist-guided individual therapy for non-responders; (Step 3) reserve dyadic sessions for cases with high relational distress, communication barriers, or caregiver–patient goal conflict, where couple-level mechanisms—not FoP per se—are the target. Health-economic evaluations should pre-specify incremental cost-effectiveness ratios (ICERs) using QALYs or validated QOL indices, include therapist time and supervision as major cost drivers, and consider budget-impact and capacity metrics (e.g., sessions delivered per FTE). In LMIC contexts, task-sharing, brief group formats, and digital delivery can expand reach without assuming cross-partner effects, which were not supported by our findings.

Our study possesses several strengths. First, it employed a robust sample of 300 dyads, surpassing the median size in recent dyadic oncology meta-analyses. Second, we used validated FoP and WHOQOL tools with demonstrated cross-cultural reliability (The WHOQOL Group, 1998). Third, structural-equation estimation with full-information maximum likelihood minimized bias from sporadic missing data. Finally, sensitivity analyses excluding advanced-stage cases or applying centered predictors confirmed model stability.

Nevertheless, limitations warrant caution. Our cross-sectional design precludes causal inference; we cannot determine whether FoP precedes and degrades QOL, whether lower QOL heightens FoP, or whether both are driven by unmeasured third variables (e.g., symptom burden, financial toxicity). Accordingly, the actor effects observed here should be interpreted as associations rather than causal pathways. Future work should use longitudinal, multi-wave designs that test cross-lagged actor and partner effects (e.g., APIM with cross-lagged paths or random-intercept cross-lagged panel models) to adjudicate directionality and to identify time windows when targeting FoP is most likely to yield QOL gains (Cohen, 1992). Self-report measures may be prone to social desirability or recall bias, although anonymous administration likely mitigated under-reporting. The sample was drawn from tertiary centers and comprised predominantly spouse caregivers, potentially limiting generalizability to rural settings or non-spousal caregivers. We also focused exclusively on global QOL; domain-specific outcomes (e.g., sexual functioning, financial toxicity) could reveal different dyadic patterns. Our use of a global QOL total may have obscured domain-specific crossover effects. Future studies should model WHOQOL-BREF domains (or comparable latent QOL factors) within APIM, incorporate repeated measures to test lagged partner influences, and consider methods that reduce role-linked social desirability (e.g., confidential digital administration, balanced domain anchors). Such designs will clarify whether FoP exerts small but clinically meaningful cross-partner effects in specific QOL domains over time. Additionally, our dyads were recruited from tertiary centres in Bangkok, and caregivers were predominantly spouses and formally employed. This urban, higher-resource context and caregiver role mix may not reflect families in provincial/rural settings, lower-income households, or dyads in which adult children, siblings, or friends are the primary caregivers. Consequently, effect sizes—particularly partner pathways—may differ where caregiving networks are more extended or where economic strain is greater. Future studies should use multi-site sampling that includes provincial hospitals and community clinics, oversample non-spousal caregivers, and stratify by socioeconomic indicators (education, income, financial toxicity). We also recommend testing moderation of actor/partner effects by caregiver role and socioeconomic status using multi-group or interaction-based APIM in adequately powered designs.

Future research should adopt multi-wave designs to model reciprocal lagged effects and identify critical windows for intervention. Mixed-methods approaches could unpack communication styles that shape FoP disclosure. Additionally, randomized controlled trials comparing individual- versus dyadic-focused FoP interventions will clarify whether tailoring to actor-only dynamics enhances efficacy. Incorporating biomarkers of stress (e.g., diurnal cortisol) may further elucidate psychophysiological pathways linking FoP to health outcomes.

In conclusion, fear of progression is a salient determinant of global quality of life for both gynecologic-cancer survivors and their caregivers, yet its impact remains largely self-contained within each partner. Screening programs and psychosocial interventions should therefore treat survivor and caregiver fears as distinct therapeutic targets. Addressing FoP in both members of the dyad—rather than presuming indirect benefits—may ultimately optimize well-being in families navigating the uncertain landscape of gynecologic cancer.

## Data Availability

The original contributions presented in the study are included in the article/supplementary material, further inquiries can be directed to the corresponding author.
